# Protective Effects of p53 Regulatory Agents Against High-LET Radiation-Induced Injury in Mice

**DOI:** 10.3389/fpubh.2020.601124

**Published:** 2020-12-03

**Authors:** Akinori Morita, Bing Wang, Kaoru Tanaka, Takanori Katsube, Masahiro Murakami, Takashi Shimokawa, Yuichi Nishiyama, Shintaro Ochi, Hidetoshi Satoh, Mitsuru Nenoi, Shin Aoki

**Affiliations:** ^1^Department of Biomedical Science and Technology, Graduate School of Biomedical Sciences, Tokushima University, Tokushima, Japan; ^2^National Institute of Radiological Sciences, National Institutes for Quantum and Radiological Science and Technology, Chiba, Japan; ^3^Department of Medicinal and Life Science, Faculty of Pharmaceutical Sciences, Tokyo University of Science, Chiba, Japan

**Keywords:** p53, radioprotector, cell death, hematopoietic syndrome, gastrointestinal syndrome

## Abstract

Radiation damage to normal tissues is one of the most serious concerns in radiation therapy, and the tolerance dose of the normal tissues limits the therapeutic dose to the patients. p53 is well known as a transcription factor closely associated with radiation-induced cell death. We recently demonstrated the protective effects of several p53 regulatory agents against low-LET X- or γ-ray-induced damage. Although it was reported that high-LET heavy ion radiation (>85 keV/μm) could cause p53-independent cell death in some cancer cell lines, whether there is any radioprotective effect of the p53 regulatory agents against the high-LET radiation injury *in vivo* is still unclear. In the present study, we verified the efficacy of these agents on bone marrow and intestinal damages induced by high-LET heavy-ion irradiation in mice. We used a carbon-beam (14 keV/μm) that was shown to induce a p53-dependent effect and an iron-beam (189 keV/μm) that was shown to induce a p53-independent effect in a previous study. Vanadate significantly improved 60-day survival rate in mice treated with total-body carbon-ion (*p* < 0.0001) or iron-ion (*p* < 0.05) irradiation, indicating its effective protection of the hematopoietic system from radiation injury after high-LET irradiation over 85 keV/μm. 5CHQ also significantly increased the survival rate after abdominal carbon-ion (*p* < 0.02), but not iron-ion irradiation, suggesting the moderate relief of the intestinal damage. These results demonstrated the effectiveness of p53 regulators on acute radiation syndrome induced by high-LET radiation.

## Introduction

In recent years, several molecular target drugs have been developed for reducing acute radiation toxicities (1–8). Although the effects of these radioprotectors have been demonstrated by experiments with low-LET radiation, we speculated that such cell death-regulating agents were also effective for protection against particle beam injury, because their radioprotective efficacies do not depend on a radical scavenging action, which is mainly effective for radiation injury caused by indirect action (9).

Although particle therapy has characteristic advantages by the superior dose distribution, the risks of radiation injury in normal tissues still determine the limits of a tolerable dose, thus cell death-regulating agents are expected as one of the non-invasive measures to reduce adverse events from occurring in organs at risk (OAR). This is especially necessary to protect OAR close to the tumor and to further improve hypofractionated regimens to increase the dose per fraction while suppressing the side effects of acute injury (10–13). In addition, although the estimated exposure dose is not quite high, it will be useful as a basic study for the development of protective agents against cosmic rays. The potential health risk in a manned interplanetary mission with a long duration up to 3 years should be reduced by proper countermeasures and the absorbed doses estimated for the largest solar particle events would be higher than 1 Gy and could reach 10 Gy in a thinly shielded spacecraft (14). Our knowledge from radiobiology indicates clearly that a dose at this latitude of even low-LET photon radiation could definitely cause significant detrimental biological effects. There are two major radiation sources of space radiation: galactic cosmic rays (GCRs) and solar particle events. GCRs consist of 85% low LET protons, 14% helium ions and 1% high atomic number and energy (HZE) particles including iron particles (15). Although these HZE particles make up only 1% of GCRs, they are high-LET particle radiations. They contribute to 21% of the estimated ionizing dose equivalent from GCR exposure during interplanetary missions in deep space and contribute significantly to the overall biological impact of cosmic rays (16).

p53 regulatory agents are expected to be applied as a normal tissue-selective radioprotective agent, because these agents selectively suppress apoptosis of OAR having normal p53 function and do not protect cancer cells that lost p53 functions (17). Furthermore, when the suppression of p53 is transient, unlike constitutive p53-knockout, p53 suppression does not promote carcinogenesis (18). On the other hand, it has been demonstrated by using tumor-derived cultured cells that particle beam-induced cell death does not show a p53 dependency on the LET of 85 keV/μm or more (19). It is unclear whether particle beam injuries in the bone marrow and intestinal epithelium, which are highly radiosensitive among OAR, are p53-dependent events.

We have already found sodium orthovanadate (vanadate) as an effective “p53 inhibitor” for bone marrow death and 5-chloro-8-quinolinol (5CHQ) as an effective “p53 modulator” for intestinal death (4, 7). Our previous work with low-LET radiations had demonstrated that vanadate is the strongest p53 inhibitor with many other biological functions; it is significantly effective against bone marrow death and partially works against intestinal death in total-body irradiation (TBI) tests, and 5CHQ is basically a p53 modulator playing important roles in modulating p53 functions, in particular, activation; it could markedly protect the intestinal death in abdominal irradiation (ABI) tests but has a weak radioprotective activity against bone marrow death in TBI tests. The difference of the spectra against acute radiation syndrome (ARS) is attributed to that p53 plays distinct roles in ARS between bone marrow death and intestinal death, in brief, p53 functions as a promoting factor that induces apoptosis in bone marrow death and as a resistance factor that prevents mitotic death in intestinal death (20). In particular, to demonstrate the protective role of p53, it is necessary to evaluate the effects of these compounds on the radiation-injured intestine using the ABI technique to avoid bone marrow death. In general, this ABI system requires more than twice the dose used in the TBI test to achieve a lethal effect. In this study, to evaluate the efficacy of these compounds, mice were irradiated systemically or abdominally with particle beams and evaluated using 60-day survival test. Regarding the radiation quality, we used a carbon-beam (14 keV/μm) that was shown to induce a p53-dependent effect and an iron-beam (189 keV/μm) that was shown to induce a p53-independent effect in a previous study (19).

## Materials and Methods

All animal experiments were performed at National Institute of Radiological Sciences (NIRS), National Institutes for Quantum and Radiological Science and Technology (QST). All experimental protocols (Experimental Animal Research Plans No. 09-1042 and No. 17-2006) involving mice were reviewed and approved by The Institutional Animal Care and Use Committee of the NIRS, QST. The experiments were performed in strict accordance with the NIRS, QST Guidelines for the Care and Use of Laboratory Animals.

### Animals

Seven weeks-old ICR strain female mice were purchased from SLC Inc. (Shizuoka, Japan) and maintained in a conventional animal facility under a 12 h light−12 h dark photoperiod and controlled temperature (22–24°C) and humidity (50 ± 5%). The animals were housed in autoclaved aluminum cages with sterilized wood chips and allowed to access standard laboratory chow (MB-1, Funabashi Farm Co., Japan) and acidified water (pH = 3.0 ± 0.2) *ad libitum*. The animals were acclimatized to the laboratory conditions for 1 week before use. To avoid possible effects from the developmental condition of the animals, 6 weeks-old mice with a significantly different body weight (more or less than the mean ± 2 SD) were omitted from this study.

### Irradiation

For high LET heavy ion irradiation, the monoenergetic ion beam of carbon and iron particles was generated and accelerated by a synchrotron, the Heavy Ion Medical Accelerator in Chiba (HIMAC) at NIRS, QST, Japan (21, 22). The beam energy was 290 MeV/nucleon and 500 MeV/nucleon for carbon- and iron-beams, and the ion beams were expanded by wobbler magnets to a 10 cm irradiation field with homogeneous irradiation dose. Samples were irradiated at the entrance (plateau) region of the ion beams corresponding to a dose averaged LET value of about 14 and 189 keV/μm, respectively. The dose rate for TBI and ABI was at about 2.7 (C-ion)/3.6 (Fe-ion) and 6.1(C-ion)/5.1(Fe-ion) Gy/min, respectively. For TBI, the mice were held in a special Lucite columnar container, which was with an outer diameter of 10 cm and 3 individual cells of the same size (each mouse in each cell). The mice were in an air-breathing condition (there were six holes 5 mm in diameter in the wall of each cell). The containers were set on the beam track and the focused 10 cm diameter carbon- or iron-beam was delivered to the animals at room temperature without anesthesia. For ABI, the animals were anesthetized by a combination anesthetic (0.3 mg/kg of medetomidine, 4.0 mg/kg of midazolam, and 5.0 mg/kg of butorphanol), fixed on a special Lucite plate using medical adhesive tapes, and then set on the beam track. The 10 cm diameter carbon- or iron-beam was further collimated to a 2 cm slit by 5 cm brass collimators and delivered locally to the whole abdominal area of the mouse. Irradiation was performed at room temperature.

### Sodium Orthovanadate (Vanadate) and 5-chloro-8-quinolinol (5CHQ)

Vanadate was purchased from Sigma-Aldrich (Sigma-Aldrich, St. Louis, MO, USA). The physiological normal saline (NS) (Otsuka Pharmaceutical Co., Ltd., Japan) was used as a solvent to make vanadate solution (2 mg/mL in NS solution). Vanadate at dose of 20 mg/kg body weight or vehicle (NS solution) was administered to the mouse 30 min before irradiation by a single intraperitoneal injection. 5CHQ was purchased from Wako Pure Chemical Industry (Wako Pure Chemical Industry, Co., Ltd., Japan) and recrystallized from ethanol for reducing possible cytotoxicity. Injection solution was prepared using dimethyl sulfoxide (DMSO) (Sigma-Aldrich, St. Louis, MO, USA) and olive oil (Wako Pure Chemical Industry, Co., Ltd., Japan) as solvents. Animals were given a single intraperitoneal injection of 5CHQ at a dose of 60 mg/kg body weight or vehicle (20% DMSO in olive oil) 30–60 min before irradiation.

### Survival Test

Survival of the animals was monitored daily up to 60 days after irradiation. For statistical analysis, log-rank test was performed when the number of animals per group was 20 or more, and chi-square test was performed when the number animals was <20 using Microsoft Excel with the add-in software Statcel 4 (OMS publisher Ltd, Saitama, Japan).

## Results

### Vanadate Is Highly Effective in Protection of Carbon-Beam TBI-Induced Death, but Not Protective Against ABI-Induced Death

**Figure 1:**
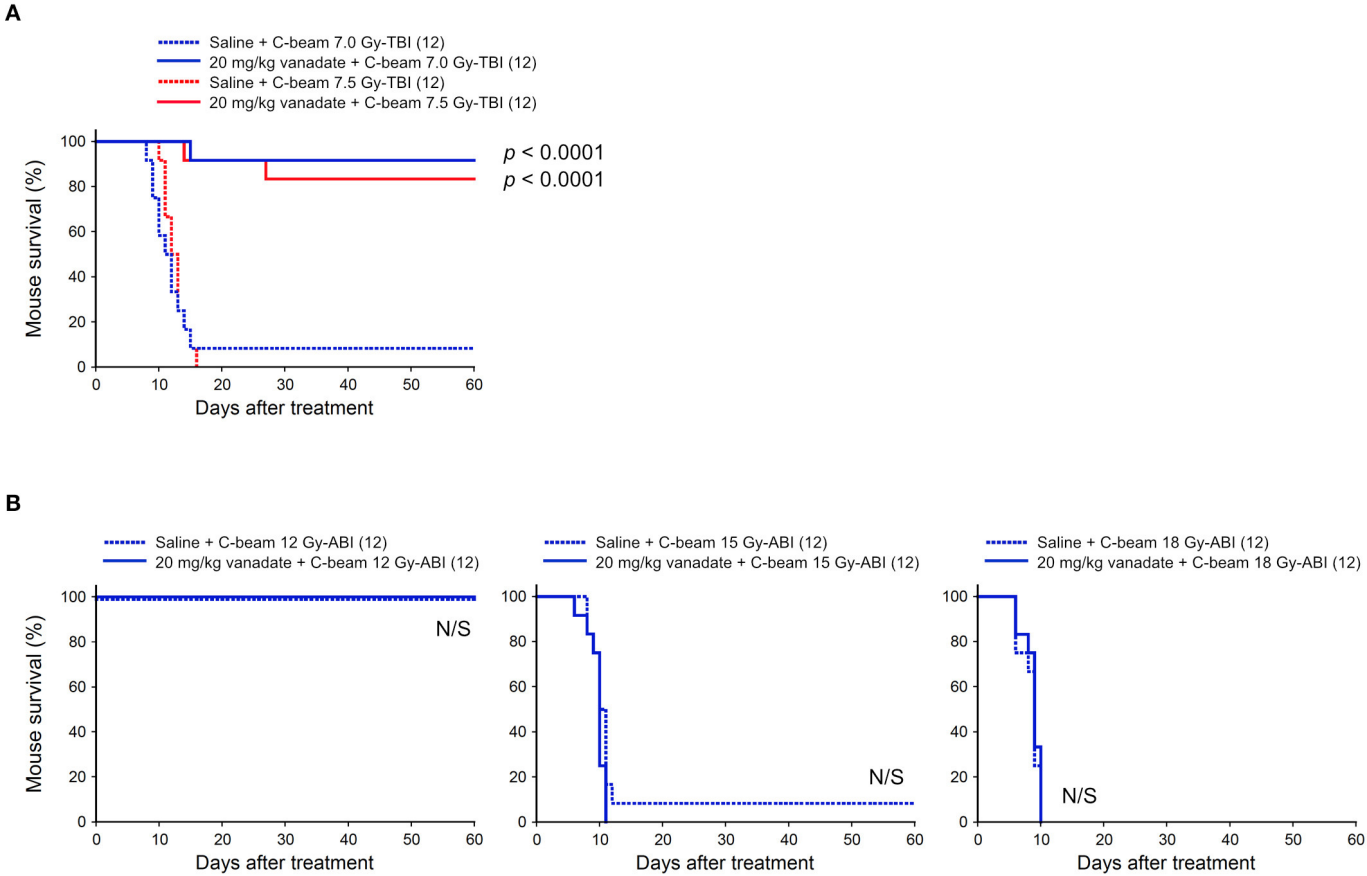
Effect of vanadate on total-body or abdominally irradiated mice with carbon-beam. ICR female mice were i.p. injected with vanadate 30 min before irradiation. Numbers in parenthesis denote the number of mice. **(A)** Sixty-day survival tests after 7.0 or 7.5 Gy-TBI of ICR mouse subgroups of vehicle alone or 20 mg/kg vanadate. By chi-square test, *p* < 0.0001, respectively. **(B)** Sixty-day survival tests after 12, 15, or 18 Gy-ABI of ICR mouse subgroups of vehicle alone or 20 mg/kg vanadate. By chi-square test, *p* = not significant (N/S), respectively.

At first, the protective effect of vanadate was examined in carbon-beam irradiation experiments (290 MeV/nucleon, mono-beam, LET 14 keV/μm). In the TBI experiments, 7.0, and 7.5 Gy were delivered to 8-week-old female ICR mice (12 mice in each irradiation group), and the survival rate was observed for 60 days (Figure 1A). In 7.0 and 7.5 Gy-TBI mice, the survival rate at 60 days was 8.3 and 0% in the vehicle-administered group, whereas the survival rate in the vanadate-administered group was 92 and 83%, respectively, showing a significant radioprotective effect of vanadate (*p* < 0.0001). In order to show the killing effect for carbon-beam ABI, we delivered 12, 15, and 18 Gy to mice, and the survival rate was observed for 60 days (Figure 1B). At the administered doses, a dose at 12 Gy-ABI was not lethal and no protective effect of vanadate was observed in 15 Gy or 18 Gy-ABI mice. These data clearly demonstrate that vanadate has a protective effect against the bone marrow death induced by carbon-beam TBI, while that it is ineffective against the intestinal death induced by carbon-beam ABI.

### Vanadate Is Also Effective in Protection of Iron-Beam TBI-Induced Death

**Figure 2:**
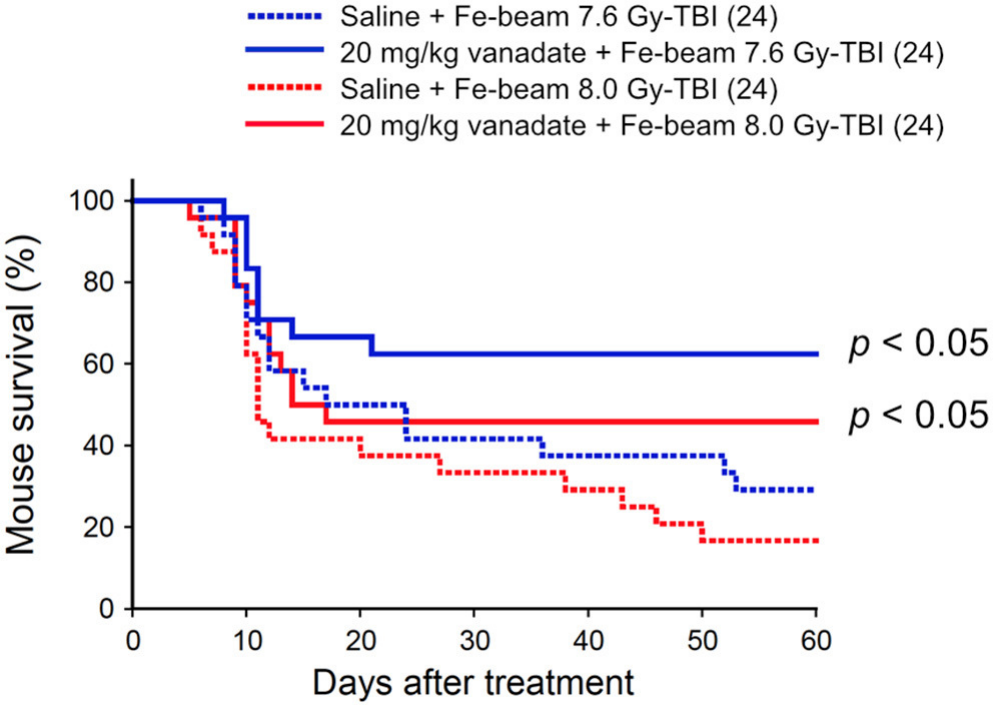
Effect of vanadate on iron-beam total-body irradiated mice. ICR female mice were i.p. injected with vanadate 30 min before irradiation. Numbers in parenthesis denote the number of mice. Sixty-day survival tests after 7.6 or 8.0 Gy-TBI of ICR mouse subgroups of vehicle alone or 20 mg/kg vanadate. By log-rank test, *p* < 0.05, respectively.

Second, the protective effect of vanadate was examined in iron-beam TBI experiments (500 MeV/nucleon, mono-beam, LET 189 keV/μm). In the TBI experiments, 7.6 and 8.0 Gy were delivered to 8-week-old female ICR mice (24 mice in each irradiation group), and the survival rate was observed for 60 days (Figure 2). In 7.6 and 8.0 Gy-TBI mice, the survival rate at 60 days was 29 and 17% in the vehicle-administered group, whereas the survival rate in the vanadate-administered group was 63 and 46%, respectively, showing a statistically significant protective effect of vanadate (*p* < 0.05), although the surviving rates were lower than those of carbon-beam experiments. These findings suggest that p53 inhibition could also suppress bone marrow death induced by high LET radiation with LET value exceeding 85 keV/μm.

### 5CHQ Is Effective in Carbon-Beam ABI-Induced Death, but Not Protective Against Carbon-Beam TBI-Induced Death and Iron-Beam ABI-Induced Death

**Figure 3:**
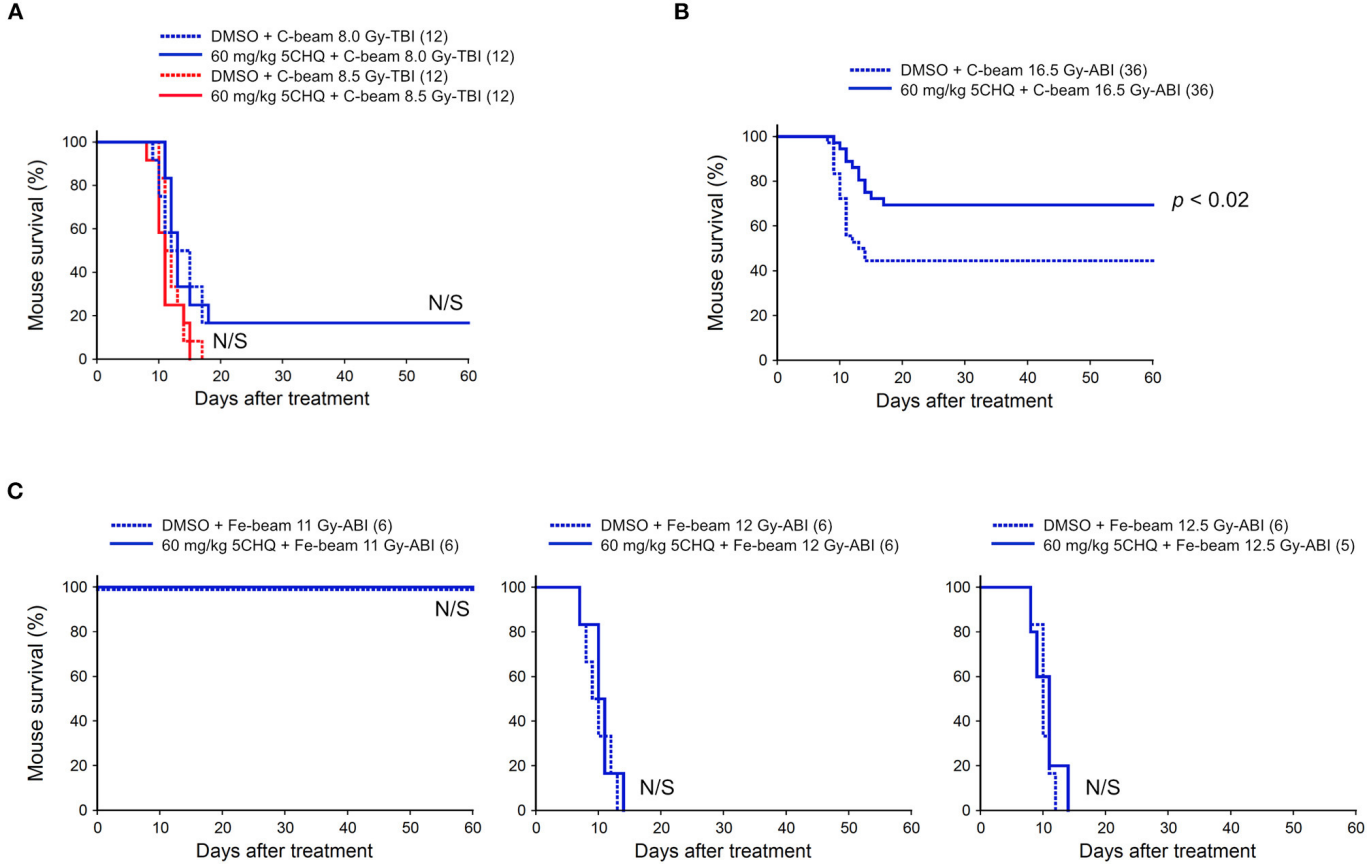
Effect of 5CHQ on total-body or abdominally irradiated mice with carbon- or iron-beam. ICR female mice were i.p. injected with 5CHQ 30–60 min before irradiation. Numbers in parenthesis denote the number of mice. **(A)** Sixty-day survival tests after 8.0 or 8.5 Gy-TBI of ICR mouse subgroups of vehicle alone or 60 mg/kg 5CHQ. By chi-square test, *p* = N/S, respectively. **(B)** Sixty-day survival tests after 16.5 Gy carbon-beam ABI of ICR mouse subgroups of vehicle alone or 60 mg/kg 5CHQ. By log-rank test, *p* < 0.02. **(C)** Sixty-day survival tests after 11, 12, or 12.5 Gy iron-beam ABI of ICR mouse subgroups of vehicle alone or 60 mg/kg 5CHQ. By chi-square test, *p* = N/S, respectively.

We also investigated the protective effect of 5CHQ, a p53 modulator which has an agonistic activity of activating radioprotective functions of p53, on the particle beam-irradiated mice. As previously reported, 5CHQ is more protective against ABI-induced intestinal death than in TBI-induced bone marrow death in mice, albeit in low-LET radiation experiments (7). Firstly, we examined the carbon-beam TBI experiments and 8.0 and 8.5 Gy were delivered to 8-week-old female ICR mice (12 mice in each irradiation group) (Figure 3A). In the TBI experiments with vehicle DMSO, a slightly higher dose than those with vehicle saline was required to achieve a lethal effect and no protective effect of 5CHQ was observed in any group of systemically irradiated mice. Next, the particle beam protective effect of 5CHQ was evaluated using abdominally irradiated mice. In the carbon-beam ABI experiment, a dose at 16.5 Gy was delivered to 8-week-old female ICR mice (36 mice in each irradiation group) (Figure 3B). As a result, the survival rate at 60 days was 44% in the vehicle-administered group, whereas the survival rate in the 5CHQ-administered group was 70%, showing a statistically significant protective effect of 5CHQ (*p* < 0.02). We also examined the protective effect of 5CHQ in the iron-beam ABI experiments (Figure 3C). At the administered doses, a dose at 11 Gy-ABI was not lethal and no protective effect of vanadate was observed in 12 Gy or 12.5 Gy-ABI mice. These data demonstrate that 5CHQ has a protective effect against the intestinal death induced by carbon-beam ABI, while that it is ineffective against that induced by iron-beam ABI.

## Discussion

In this study, we evaluated the protective activity of p53 regulatory agents against bone marrow death by particle beam TBI and intestinal death by particle beam ABI in the mouse models. At first, considering that iron-beam causes more severe damage to cells than carbon-beam at the same dose and saving animal lives whenever potentially applicable, tests for “vanadate vs. Fe-beam ABI” and “5CHQ vs. Fe-beam TBI” had not been performed as these agents showed ineffective against carbon-beam in the corresponding tests (Figures 1B, 3A).

Considering that vanadate inhibits p53, the results that vanadate was highly effective in the TBI experiments, and no protective effect was observed in the ABI experiments are appropriate. The ineffectiveness of vanadate against intestinal death by ABI was also confirmed by low-LET X-ray ABI experiments (Nishiyama et al., manuscript in preparation). Because these results are consistent with reports of p53's tissue response that promotes cell death in the bone marrow and is protective in the intestine upon radiation injury (20). Of note, when comparing the mouse survival after carbon- and iron-beam injuries, iron-beam injury is characterized by the late occurrence of deaths even 30–60 days after irradiation (Figures 1, 2). This delayed iron-beam damage was suppressed by vanadate. The cause of delayed death is not clear, it may not be due to the bone marrow suppression when taking into account the short life span of the hematopoietic lineage cells. As such a late fatal radiation injury, radiation angiopathy caused by a delayed cell death of endothelial cells is well known (23). Further studies are needed to explore the cause of death using different approaches such as pathological examinations or bone marrow transplantation. In any case, it is clear that there are cell populations that can avoid the fate of cell death even after exposure to particle beams with an LET value higher than 85 keV/μm by pharmaceutically regulating cell death machinery. The dense lesions induced by these high-LET radiations are considered to be too severe to be regulated by p53 (19). Our data demonstrate that even in such a circumstance, at least some cells are rescued by p53 regulatory agent. It is also considered that vanadate has various activities such as protein tyrosine phosphatase inhibition (24, 25) and activates radioresistant factors other than p53 to exert its radioprotective activity. We will focus our next research on solving these research issues.

As shown in Figure 3, 5CHQ was effective against carbon-beam intestinal injury, but ineffective against carbon-beam bone marrow injury and iron-beam intestinal injury. The ineffectiveness of 5CHQ for iron-beam intestinal injury may suggest its activity was not strong enough as a p53 modulator rather than the limit of therapeutic strategies for suppressing intestinal death by p53 modulators. The discovery of a p53 modulator with a stronger activity than 5CHQ might improve the protective effect. Considering that the current clinical heavy ion radiotherapy is performed using carbon beams for the treatment of tumors in the pelvic region, suppression of radiation intestinal disorders by application of p53 modulators is expected to be a useful therapeutic strategy.

## Data Availability Statement

The raw data supporting the conclusions of this article will be made available by the authors, without undue reservation.

## Ethics Statement

The animal study was reviewed and approved by Animal Care and Use Committee of the National Institute of Radiological Sciences, National Institutes for Quantum and Radiological Science and Technology.

## Author Contributions

AM, BW, YN, TS, and MN designed research. AM, BW, KT, TK, MM, and SO performed research. AM, BW, and SO analyzed data. HS and SA contributed a purification of 5CHQ by recrystallization. AM, BW, YN, TS, and MN wrote the manuscript. All authors contributed to manuscript revision, read, and approved the submitted version.

## Conflict of Interest

The authors declare that the research was conducted in the absence of any commercial or financial relationships that could be construed as a potential conflict of interest.
